# Impact of simulation debriefing structure on knowledge and skill acquisition for postgraduate critical care nursing students: three-phase vs. multiphase

**DOI:** 10.1186/s12912-022-01100-z

**Published:** 2022-11-22

**Authors:** Jefferson Garcia Guerrero, Grace Medalyn Tungpalan-Castro, Minerva Pingue-Raguini

**Affiliations:** Nursing Department, Fakeeh College for Medical Sciences, Abdul Wahab Naib Al Haram, Al-Hamra’a, 23323 Jeddah, Saudi Arabia

**Keywords:** High fidelity simulation, Debriefing structure, Constructive criticism, Knowledge acquisition, Skills development, Clinical competence

## Abstract

**Background:**

Simulation is part of the training provided to nurses enrolled in the master’s degree for critical care nursing programmes at our institution. Although the students are practicing nurses, many still make mistakes when performing nursing procedures related to critical care during simulation sessions, and these mistakes must be addressed during the debriefing session. The aim of the study is to compare the knowledge and skills acquired by groups of postgraduate critical care nursing students who were exposed to high-fidelity simulation (HFS) by using different debriefing structures.

**Methods:**

A quasi-experimental crossover design was utilised during the post-tests and objective structured clinical examinations (OSCEs). The students were divided into two groups: one was exposed to HFS with a 3-phase debriefing, and the other was exposed to HFS with a multiphase debriefing. Both groups involved facilitator-guided and video-assisted debriefings.

**Results:**

Overall, the post-test scores (*p-value*: Phase 1 = 0.001 and Phase 2 = 0.000) and post-OSCE scores (*p-value*: Phase 1 = 0.002 and Phase 2 = 0.002) support that the group of postgraduate students who underwent HFS with a multiphase debriefing structure gained significantly higher scores compared to the group who underwent HFS with a 3-phase debriefing structure.

**Conclusion:**

Debriefing is a critical component of successful simulation. Learning requires assessment that creates constructive criticism based on feedback and reflection. A multiphase debriefing structure, specifically the healthcare simulation after-action review, provides a significant advantage for knowledge and skills acquisition.

## Background

Simulation is part of the clinical practicum of nurses enrolled in master’s degrees in critical care nursing programmes at Fakeeh College for Medical Sciences in Jeddah, Saudi Arabia. It enhances their current knowledge and skills in caring for critically ill patients. Knowledge and skills acquisition is crucial for postgraduate critical care nursing students because it is a crucial element of attaining competence in the field of critical care. Moreover, it nurtures personal and professional accountability. Therefore, appropriate knowledge and skills promote quality care and patient safety. In the process of learning, recognition of shortcomings and identifying improvements are pivotal aspects of knowledge acquisition and development. In the healthcare field, one of the most important strategies for ensuring that nursing students and practitioners have cultivated knowledge from simulation-based training is the application of the debriefing structure. This implies that a specific and active intervention process (primary goals of debriefing) is key to reviewing individual and team performance during simulation training. From these discussions, learners can contemplate their actions that will encourage and integrate better performance in actual medical situations. This study compared three-phase and multiphase debriefing structures because the simulation facilitators in the Clinical Skills and Simulation Center (CSSC) are not unified on which debriefing structure will be used, although they were trained on both. Identifying the best debriefing structure that produces exceptional outcomes in knowledge and skills acquisition of nurses will help achieve the goal of the simulation.

Simulation is a technique that imitates real-life situations. It is utilised in several contexts, such as an educational process for students to implement learned skills and re-enact performances before the actual delivery to the healthcare system [1, 2]. After the simulation, a follow-up procedure is needed for the reflective process of the students⁠ through debriefing procedures. Students can reflect, contemplate, and associate their physical performances during the simulation to fully grasp the cognitive interpretation of the scenario during the debriefing process [2, 3]. Generally, a debriefing conducted right after a simulation scenario is more effective than one during the simulation in terms of knowledge and confidence [4, 5]. Moreover, debriefing should be based on a structured framework; however, researchers disagree over which framework is most effective [6–9].

In the process of simulated learning experiences, debriefing emerged as the foundation of the learning experience’s success [2]. Hence, debriefing can be a significant factor in achieving efficiency in experiential learning [10–12]. Following a simulated or real-life scenario, debriefing allows students to reflect, explore, and provide more appropriate actions and clinical reasoning patterns to foster improved quality in the healthcare system [12].

Receiving structured debriefing is identified as a fundamental part of attaining effective learning [13–15]. Incorporating the debriefing process into simulation-based training provides more opportunities for enhanced learning and improves the participants’ awareness of their actions and effectiveness. As a structured process, debriefing develops students’ learning capacity; it stimulates the transfer of knowledge and improves technical and behavioural skills. Additionally, debriefing offers best practices to be integrated into the participants’ professional role to achieve safe and quality healthcare [16–18].

The high-fidelity simulation (HFS) that integrates debriefing is accepted as an essential component of nursing education. The training provided by these two learning processes is an essential tool to achieve advancements in the nurses’ performance in their traditional and professional roles [19–22]. During training, the practice of self-reflection skills becomes a beneficial aspect for aspiring simulation facilitators. It improves their capacity to explore emotions, allowing them to establish techniques in communication and improve their problem-solving skills to form judgements [23]. However, owing to a lack of evidence in nursing literature, limited support is available for Advanced Practice Registered Nurses or Acute Care Nurse Practitioner education to prove the efficacy of HFS and debriefing practices [19, 21, 24]. Furthermore, despite clear evidence supporting the use of simulation in nursing education, there is little confirmation regarding the most effective debriefing strategy or model for use in debriefing simulations designed for nurses in post-graduate critical care nursing programmes.

In HFS, an essential aspect of learning is the process of debriefing; thus, the core of the literature on HFS focuses predominantly on the process of debriefing and the reflection on action learning [25–28]. To attain the standards of debriefing, the International Nursing Association for Clinical Simulation and Learning highlights the currently available framework, such as gather, analyse, summarise (GAS); debriefing with good judgement; promoting excellence and reflective learning in simulation; debriefing for meaningful learning; plus-delta; 3D model of debriefing; and the outcome-present state test model of clinical reasoning. The association also set a fundamental criterion to follow: (1) qualified person(s) who facilitate a debrief must be capable and have adequate knowledge for conducting the debrief; (2) the debrief should be operated in a setting that is favourable to learning and promotes confidentiality, reliability, free-flow of communication, self-awareness, personal analysis, assessment, healthy criticism, and intrapersonal skills; (3) facilitator(s) must show commitment in providing sufficient concentrated attention during the debrief to achieve its efficacy after the simulation-based experience; (4) the foundation of the debrief should be based on a theoretical framework that represents its purpose; and (5) there should be harmony between the debrief, objectives, and outcomes of the simulation training [17].

Approximately 10 million Saudi Riyals were invested by the [institution name blinded for peer review] to construct the CSSC in March 2019. The facility has the mandate to promote the development of clinical performance and enhanced competence among undergraduate and postgraduate nursing students in compliance with the national and international standards of nursing practice. This study aimed to compare the knowledge and skills acquired by groups of postgraduate critical care nursing students exposed to HFS utilising different debriefing structures (three-phase and multiphase).

## Methods

This study utilised a quasi-experimental crossover design to compare knowledge and skills acquired by the groups of postgraduate critical care nursing students exposed to HFS utilising different debriefing structures, such as three-phase and multiphase. The crossover design was utilised to establish which debriefing structure was more effective for knowledge and skills acquisition while the students experienced both structures. The design also excluded biases that might occur owing to individual competency within the allocated group [29].

The postgraduate students were divided into two groups: one was exposed to HFS with the three-phase debriefing. The GAS model, which included the phases of gathering, analysing, and summarising [30], was used for this group. By utilising this structure, the first phase (gather) created a shared mental model by providing a restatement of the simulation event. The second phase (analysis) was dedicated to the analysis of the actions during the simulation and learner-centred reflections. The final phase (summary) provided a review of the lessons learned and ensured that all teaching points and key learning objectives had been covered [31–33].

The other group was exposed to HFS with multiphase debriefing. The healthcare simulation after-action review (AAR) was applied to this group. Using this structure, the debriefing was conducted in seven phases represented by the acronym DEBRIEF. These phases were as follows: defining rules, explaining learning objectives, benchmarking performance, reviewing expected actions, identifying what occurred, examining why things occurred the way they did, and formalising learning [34]. Additionally, both debriefing structures were facilitator-guided and video-assisted.

Prior to the study, intensive training on technical preparation and operation was already conducted for nursing faculty members, including the researchers who conducted the current study on the available high-fidelity simulators of the institution. The training also addressed simulation clinical scenario (SCE) development, how to utilise CAE LearningSpace to run simulation scenarios, and simulation process and workshops for debriefing using the following structures: the GAS model and Healthcare Simulation AAR framework. However, during the study, only one facilitator was assigned to conduct all aspects of the simulation (pre-briefing, simulation scenario, and debriefing) for each scenario conducted. All SCEs, including objectives and stations, were formulated by the facilitators with the help of the clinical instructors and nurse preceptors in the intensive care units of the college-based hospital. They were further reviewed by the faculty members specialising in critical care nursing. Moreover, the debriefings were standardised.

### Participants

Postgraduate students currently enrolled in the critical care nursing practicum course were recruited for the study. The participants were both male and female, with an age range of 27–45 years, and had bachelor of science in nursing degrees with licenses to practice nursing in Saudi Arabia. All participants were working in either government or private tertiary hospitals in Saudi Arabia as staff nurses, nurse educators, and nurse managers in the critical care unit. They had more than 3 years of experience in critical care and comprised Saudi and non-Saudi nationals.

Thirty-seven postgraduate students were enrolled in the critical care nursing practicum course, and all were given the opportunity to participate in the study. The researchers selected a total of 30 participants, 15 of whom were randomly selected for Group A and the other 15 for Group B. Their student ID numbers were written on paper and selected through a lottery draw. The researchers included 30 participants out of 37 because 4 of them refused to participate, and 3 others did not complete all phases of the HFS sessions owing to availability issues and other personal circumstances. Therefore, complete enumeration was applied.

The details and purpose of the study were explained to the participants, and informed consent was obtained in writing before the study commenced. These students were exposed to HFS sessions but utilised different debriefing structures, such as the three-phase structure that follows the GAS model and the multiphase structure using the Healthcare Simulation AAR framework.

### Data collection procedure

Simulation sessions were divided into three phases: pre-briefing, simulation scenario, and debriefing. Phase 1 of the HFS exposure was conducted with three scenarios, each conducted on different days (with CAE iStan and CAE Apollo): (Sim1) managing angina and myocardial infarction, (Sim2) burns and spinal shock management, and (Sim3) trauma for fractures and amputations. Group A underwent the three phases of simulation (pre-briefing, simulation scenarios, and debriefing) using the GAS model. Group B underwent simulation sessions (including pre-briefing, simulation scenarios, and debriefing) utilising the ‘Healthcare Simulation AAR’ framework.

Phase 2 of HFS exposure was conducted with another three scenarios on different days (with CAE iStan and CAE Apollo): (Sim4) managing pneumonia with septic shock, (Sim5) managing pulseless ventricular tachycardia with defibrillation, and (Sim6) trauma for managing chest stab wounds. This time, Group A underwent simulation sessions (including pre-briefing, simulation scenarios, and debriefing) utilising the Healthcare Simulation AAR framework. Group B underwent the three phases of simulation sessions (pre-briefing, simulation scenarios, and debriefing) using the GAS model. All scenarios with the identified objectives were applied during Phases 1 and 2 of the HFS sessions (see Table 1). Furthermore, the six scenarios were created and selected based on the listed competencies in the course specifications. The shifting of debriefing structures applied to the group of participants during Phases 1 and 2 was considered to challenge the impact of three-phase and multiphase to both groups of participants in a given scenario.

**Table 1:** HFS sessions and durations of pre-briefing, scenario, and debriefing

		Duration		
**Sessions**	**Scenarios**	**Pre-briefing**	**Scenario**	**Debriefing**
*Phase 1*				
** Sim1**	Managing angina and myocardial infarctions	20–30 min	8–10 min	20–35 min
** Sim2**	Burns and spinal shock management	20–30 min	8–10 min	20–35 min
** Sim3**	Trauma: fractures and amputations	20–30 min	8–10 min	20–35 min
*Phase 2*				
** Sim4**	Managing pneumonia with septic shock	20–30 min	8–10 min	20–35 min
** Sim5**	Managing pulseless ventricular tachycardia with defibrillation	20–30 min	8–10 min	20–35 min
** Sim6**	Trauma: managing chest stab wounds	20–30 min	8–10 min	20–35 min

Before the debriefing, the facilitator reviewed the participants’ performance through the recorded video and organised their thoughts and observations. Thereafter, the facilitator went through the steps of the debriefing structure (GAS model and Healthcare Simulation AAR framework) and rehearsed what they discussed. All materials (e.g. flipped charts, television, computer, and videos) were prepared and tested.

During the debriefing phase, following the GAS debriefing model, facilitators asked each participant to share their experience through a narrative; they were also asked to provide their emotional state and reaction while undergoing simulation sessions. Following the group sharing, facilitators reviewed and recorded the details of the sessions, provided feedback about the participants’ performances, and provided reports on their observations highlighting the correct and incorrect steps that were executed by participants. To challenge the participants’ critical thinking process, facilitators asked questions while encouraging reflection and administering redirection. During the final part of the debriefing, participants were tasked with providing a list of actions they felt were efficient and adequate for the success of the simulation sessions. From this list, participants were able to identify, discuss, and rethink the necessary actions required to improve their performance in future simulations or actual healthcare settings. In the Healthcare Simulation AAR framework, the facilitator first emphasised that the debriefing is a discussion of participants’ experiences during the simulation and not a lecture or critique; hence, all participants were encouraged to share their experiences. It was also emphasised that the purpose of debriefing was to learn from each other’s experiences. Second, the facilitator reviewed the learning objectives of the scenario with the participants. Third, they benchmarked participants’ performance and actions based on the standards written in the references used in the scenario. Fourth, they gave a brief explanation of the simulation scenario and discussed the actions that were expected from participants. Fifth, they discussed observations on what was actually happening during the scenario. Sixth, they asked a volunteer from the participants to recap what was happening during the simulation and identify the correct and incorrect actions taken to fill the performance gaps. Lastly, each participant was asked to answer the following questions briefly: ‘What went well in the scenario?’; ‘What did not go well?’; and ‘What would you do next time if you are faced with a similar situation in real life?’ These questions were based on the theory of experiential learning [35].

The knowledge acquired by participants was assessed with a post-test in each conducted scenario. These tests were situational and required critical thinking. Each test comprised 25 multiple-choice questions (MCQs) with five choices: 1 point for each question, 5 essay (open-ended) questions, and 5 points per question, for a total of 50 points on each simulation session. The test questions were prepared by the faculty members and clinical preceptors involved in the simulation training. Critical care nursing books and scenarios were used as a reference to develop questions. The test was then sent, along with a copy of the simulation scenarios, to two critical care nursing experts who checked its appropriateness and validity. A pilot test was conducted on 10 clinical preceptors from the base hospital to ensure that the exam duration was less than 45 min, and the item analysis was calculated (15 moderate MCQ questions, 3 difficult MCQ questions, 3 challenging MCQ questions, 2 moderate essay questions, 1 difficult essay question, and 1 challenging essay question) for each simulation scenario performed. The post-test reliability for each simulation scenario was tested using Cronbach’s alpha (*Sim1*: 0.86, *Sim2*: 0.89, *Sim3*: 0.88, *Sim4*: 0.82, *Sim5*: 0.87, and *Sim6*: 0.91). According to Taber, an alpha value ranging from 0.73 to 0.95 indicates high reliability [36]. The test was encoded and overseen by a computer-based exam software known as the Speedwell system.

Furthermore, the skills acquired by participants were assessed by conducting objective structured clinical examination (OSCE) in every scenario. The course coordinators and clinical instructors prepared and reviewed all the objectives and scenarios, with a list of equipment needed in each station and procedural rubrics based on the references and scenarios used during the HFS sessions. The OSCE rubrics were validated by the same critical care nursing experts who validated the post-tests. The OSCE stations were set up appropriately, according to the blueprint. All rubrics used a four-point Likert-type scale: 3 (done correctly), 2 (done incompletely), 1 (done incorrectly), and 0 (not done); the indicators used were dependent on the procedure. Raters and students were instructed regarding their roles in the OSCE task. Station numbers were assigned to laboratories. All the required equipment was arranged accordingly to avoid confusion. The standardised patients were briefed and oriented on their roles during the examination. Raters comprised the clinical instructors of Fakeeh College for Medical Sciences, while the invited preceptors were staff nurses of Dr. Soliman Fakeeh Hospital. Every OSCE station had two raters, and they were blinded to the student groups. The post-OSCE reliability was tested using Kappa coefficients (*Sim1*: 0.91, *Sim2*: 0.87, *Sim3*: 0.91, *Sim4*: 0.86, *Sim5*: 0.91, and *Sim6*: 0.89). According to Cohen, a Kappa result ranging from 0.81 to 1.00 indicates almost perfect agreement [37, 38]. The OSCE checklist was also encoded in the institution’s Speedwell system.

### Data analysis

The post-test and OSCE scores were automatically calculated and generated in the Speedwell system. The test scores of all students were downloaded and summarised for data analysis. The collected data were encoded and analysed using IBM SPSS 2020 software, while mean and standard deviations were used to summarise the data. The t-test was used to compare the post-test scores and OSCE scores for the group of postgraduates exposed to HFS by utilising different debriefing structures. A normality test was performed as a basis to utilise parametric analysis for comparing the two groups of participants. The post-test had a skewness value of 1.85 and a standard error (SE) of 0.68, whereas the post-OSCE had a skewness value of -0.41 with an SE of 0.12, accordingly.

### Ethical considerations

The purpose of the study was explained to the postgraduate critical care nursing students, who agreed to participate before the study commenced. Ethical approval for this study was obtained from the Fakeeh College for Medical Sciences Institutional Review Board (Approval No. 288/IRB), and all methods were performed in accordance with the Declaration of Helsinki. Written informed consent was obtained from all participants.

## Results

The participants comprised 9 men and 21 women, with an age range of 27–45 years. Each had a Bachelor of Science in Nursing degree with a license to practice nursing in Saudi Arabia. Sixteen participants were working in government hospitals, whereas the rest were working in private hospitals in Saudi Arabia as staff nurses, nurse educators, and nurse managers in the critical care unit. They each had more than 3 years of experience in critical care and comprised 17 and 13 Saudis and non-Saudi nationals, respectively.

Table 2 shows the comparison of participants’ acquired knowledge through the post-test scores from the two phases of simulation sessions (Phase 1: Sim1, Sim2, and Sim3; Phase 2: Sim4, Sim5, and Sim6) from the two groups of postgraduate critical care nursing students: Group A who experienced HFS utilising the three-phase debriefing structure (GAS model) and Group B who experienced HFS utilising the multiphase debriefing structure (Healthcare Simulation AAR framework) during Phase 1; and Group A who experienced HFS utilising the multiphase debriefing structure (Healthcare Simulation AAR framework) and Group B who experienced HFS using the three-phase debriefing structure (GAS model) during Phase 2.

**Table 2:** Comparison of participants’ acquired knowledge through the post-test scores

***Phase 1***									
Scores	Groups	*Sim 1*	*Sim 2*	*Sim 3*	Mean	SD	Mean Difference	*p-value*	Difference
* Post-test*	Group A (three-phase debriefing structure)	78.50	81.25	79.75	79.83	1.37	11.00	0.001	Significant
	Group B (multiphase debriefing structure)	89.75	92.75	90.00	90.83	1.66			
***Phase 2***									
Scores	Groups	*Sim 4*	*Sim 5*	*Sim 6*	Mean	SD	Mean Difference	*p-value*	Difference
* Post-test*	Group A (multiphase debriefing structure)	91.50	90.00	90.75	90.75	0.75	-9.58	0.000	Significant
	Group B (three-phase debriefing structure)	82.00	80.50	81.00	81.1	0.76			

In Phase 1, the mean of the post-test scores of Group A in the three-simulation session was 79.83%, with a standard deviation (SD) of 1.37%, which is significantly lower than the post-test scores of Group B, with a mean of 90.83% and an SD of 1.66% (a mean difference of 11.00%, supported by a *p*-value of 0.001). However, in Phase 2, the mean of the post-test scores of Group A in the three-simulation session was 90.75%, with an SD of 0.75%, which is significantly higher than the post-test scores of Group B, with a mean of 81.1% and an SD of 0.75% (a mean difference of -9.58%, supported by a *p*-value of 0.000) (see Table 2).

Table 3 shows the comparison of the skills developed by participants through the post-OSCE scores from the two phases of simulation sessions (Phase 1: Sim1, Sim2, and Sim3; Phase 2: Sim4, Sim5, and Sim6) from the two groups of postgraduate critical care nursing students: Group A who experienced HFS utilising the three-phase debriefing structure (GAS model) and Group B who experienced HFS utilising the multiphase debriefing structure (Healthcare Simulation AAR framework) during Phase 1; and Group A who experienced HFS utilising the multiphase debriefing structure (Healthcare Simulation AAR framework) and group B who experienced HFS using the three-phase debriefing structure (GAS model) during Phase 2.

**Table 3:** Comparison of the skills developed by participants through the post-OSCE

***Phase 1***									
Scores	Groups	*Sim 1*	*Sim 2*	*Sim 3*	Mean	SD	Mean Difference	*p-value*	Difference
* Post-OSCE*	Group A (3-phase debriefing structure)	85.31	87.50	89.37	87.39	2.03	9.51	0.002	Significant
	Group B (multiphase debriefing structure)	95.43	97.18	98.12	96.91	1.36			
***Phase 2***									
Scores	Groups	*Sim 4*	*Sim 5*	*Sim 6*	Mean	SD	Mean Difference	*p-value*	Difference
* Post-OSCE*	Group A (multiphase debriefing structure)	96.87	97.50	98.75	97.70	0.95	-9.17	0.002	Significant
	Group B (three-phase debriefing structure)	87.18	88.12	90.31	88.53	1.60			

In Phase 1, the mean of the post-OSCE scores of Group A in the three-simulation session was 87.39%, with an SD of 2.03%, which is significantly lower than the post-OSCE scores of Group B, with a mean of 96.91% and an SD of 1.36% (a mean difference of 9.51%, supported by a *p*-value of 0.002). However, in Phase 2, the mean of the post-OSCE scores of Group A in the three-simulation session was 97.70%, with an SD of 0.95%, which is significantly higher than the post-OSCE scores of Group B, with a mean of 88.53% and an SD of 1.60% (a mean difference of -9.17%, supported by a *p*-value of 0.002) (see Table 3).

## Discussion

This study utilised a quasi-experimental research design during the post-tests and OSCEs of a group of postgraduate critical care nursing students exposed to HFS with three-phase and multiphase debriefing structures. The results indicate that the knowledge acquired by the students, as observed in their post-test scores and the skills they developed from the OSCEs scores after exposure to HFS with the multiphase debriefing structure (specifically, healthcare simulation AAR), provided a great advantage. The use of the multiphase debriefing structure showed better results in terms of students’ acquired knowledge and skills compared with the three-phase debriefing structure owing to its comprehensive and detailed sequential approach. In multiphase debriefing, the conversational structures were expanded with its seven sequential steps that allowed specific emphasis on key themes and supported the debriefing conversation. According to Piaget, individuals generate knowledge through the interaction between experiences and ideas [10]. Piaget’s view of constructivism offers inspiration for fundamental constructivism because he believed that the individual is at the centre of the knowledge construction and acquisition process. Furthermore, an AAR delivers a means to witness and review actions commenced in response to an actual situation or scenario [39] and provides an incomparable opportunity for reflection and collective learning [40].

It is common knowledge that experience alone is insufficient in the learning process. This is why the use of HFS is insufficient in nursing education, and the integration of self-reflection is vital. Ideally, educators or simulators who use and perform HFS should possess adequate knowledge and have undergone sufficient training to guide students through their reflective learning process [31, 32]. The goal of reflection is to tap into the consciousness of learners and contemplate their given actions; with this perspective, they can fully understand their pre-existing knowledge and acquire additional information, abilities, and attitudes [17, 41–43].

With HFS training being a pivotal process in nursing education, students were able to apply their proficiencies in an imitated setting of what can occur during an actual clinical scenario. The HFS training and multiphase debriefing structure heightened and challenged the students’ clinical reasoning, decision-making skills, and judgements. This led to a higher chance of gaining new knowledge and its transferability to actual nursing care within the healthcare facility. Essential to the process of simulation is the method of debriefing, where students were encouraged to examine and assess the ‘know-what’, ‘know-how’, and ‘know-why’ during their HFS training.

Simulation is a valuable strategy in teaching that promotes a higher and more diverse level of learning through the evaluation of clinical skills for nursing and midwifery education, including undergraduate, postgraduate, and lifelong education [44, 45]. Moreover, the fashioned active pedagogical strategy of simulation training is beneficial for the consolidation and knowledge of students. It promotes progress in technical and relational skills and pushes students to construct rules and habits of critical thinking and reflection, which is a necessary mindset for competent professionals [44].

One of the key concepts of Piaget’s Constructivist Theory underlines that learning is a cognitive process; learners develop thinking through trial and error, forming knowledge through experiences [46, 47]. Additionally, in the process of learning, the teacher or facilitator guides the integration of new knowledge into its existing framework. The simulation offers this type of constructivism by ensuring that students can transfer their formed knowledge and skills based on experience into actual clinical settings. Moreover, simulation fosters collaboration among the students and facilitators [47, 48]. This is strengthened and justified by the debriefing process, in which reflection is conducted to identify and create a particular and necessary change to enhance subsequent action [47, 49, 50].

Through simulation, students acquired the possibility of increasing their knowledge and skills by applying previous learning to gain new proficiency; their studied theoretical notions will be put into direct practice. Finally, through debriefing, students were able to reflect and assess the actions they performed during the simulation to come up with better and more appropriate action plans for application in real clinical settings [47, 49, 50]. Simulation is a vital educational tool for nursing students to improve their critical thinking and enhance their clinical reasoning in complex care situations [47, 51].

### Limitations

This study had a small sample size and involved only one setting and location due to the coronavirus disease 2019 restrictions regarding face-to-face interactions. The use of multiphase debriefing is preferred by the simulation facilitators considering their level of experience. Thus, to determine the generalisability of the study findings, additional studies with a larger sample in different settings are recommended. Furthermore, pre-test and pre-OSCE were not conducted since it was the first time the students were exposed to HFS and debriefing. Additionally, the content validity index of post-tests and post-OSCE rubrics were not calculated.

## Conclusion

In conclusion, debriefing is a critical component of successful simulations for nursing students and practitioners. The participants’ reflection on their actions during the simulation training offers them a safe environment to determine mistakes they may have made, think of more suitable actions in response to this, change their behaviours, and collaborate with their facilitators to improve their performance. Learning requires assessment, which creates constructive criticism centred on reflection based on their action. Moreover, the use of a multiphase debriefing structure, specifically the healthcare simulation AAR, provides a great advantage for knowledge and skills acquisition.

### Recommendations

The study recommends a reassessment of the same participants after a period of 3 to 6 months to identify transferability and learning retention. Further experimental studies on many participants regarding debriefing timing, facilitation, and testing effectiveness of the currently available structures of debriefing must be conducted. It is necessary to conduct intensive training programmes for nursing faculty members when facilitating debriefing to apply the best practices and enable reflective discussions after performing simulation scenarios. Creating a safe environment for the learners is conducive to conducting these debriefing sessions.

## Data Availability

The datasets used and analysed during the current study are available from the corresponding author upon reasonable request.

## Abbreviations

HFS: High-fidelity simulation
OSCEs: Objective structured clinical examinations
AAR: After-action review
GAS: Gather, analyse, summarise
CSSC: Clinical Skills and Simulation Center
MCQs: Multiple-choice questions
SCE: Simulation clinical scenario
SE: Standard error
SD: Standard deviation
